# Self-reported sitting time and physical activity: interactive associations with mental well-being and productivity in office employees

**DOI:** 10.1186/s12889-015-1447-5

**Published:** 2015-01-31

**Authors:** Anna Puig-Ribera, Iván Martínez-Lemos, Maria Giné-Garriga, Ángel Manuel González-Suárez, Judit Bort-Roig, Jesús Fortuño, Laura Muñoz-Ortiz, Jim McKenna, Nicholas D Gilson

**Affiliations:** Departament de Ciències de l’Activitat Física, Universitat de Vic, Barcelona, Spain; Universidad de Vigo, Vigo, Spain; Physical Activity and Sport Sciences Department, FPCEE Blanquerna, Universitat Ramon Llull, Barcelona, Spain; Departamento de Educación Física y Deportiva, Universidad del País Vasco, Leioa, Spain; Physical Therapy Department, FCS Blanquerna, Universitat Ramon Llull. Esport3 Association, Barcelona, Spain; Unitat de Suport a la Recerca Metropolitana Nord, Institut Universitari d’Investigació en Atenció Primària Jordi Gol (IDIAP Jordi Gol), Santa Coloma de Gramenet, Barcelona, Spain; School of Sport, Leeds Metropolitan University, Leeds, UK; School of Human Movement Studies, University of Queensland, St Lucia, Australia; Departament de Ciències de l’Activitat Física, Universitat de Vic, Carrer de la Sagrada Família, 7, 08500 Vic (Barcelona), Catalonia Spain

**Keywords:** Sitting time, Physical activity, Mental well-being, Work productivity, Office employees

## Abstract

**Background:**

Little is known about how sitting time, alone or in combination with markers of physical activity (PA), influences mental well-being and work productivity. Given the need to develop workplace PA interventions that target employees’ health related efficiency outcomes; this study examined the associations between self-reported sitting time, PA, mental well-being and work productivity in office employees.

**Methods:**

Descriptive cross-sectional study. Spanish university office employees (n = 557) completed a survey measuring socio-demographics, total and domain specific (work and travel) self-reported sitting time, PA (International Physical Activity Questionnaire short version), mental well-being (Warwick-Edinburg Mental Well-Being Scale) and work productivity (Work Limitations Questionnaire). Multivariate linear regression analyses determined associations between the main variables adjusted for gender, age, body mass index and occupation. PA levels (low, moderate and high) were introduced into the model to examine interactive associations.

**Results:**

Higher volumes of PA were related to higher mental well-being, work productivity and spending less time sitting at work, throughout the working day and travelling during the week, including the weekends (p < 0.05). Greater levels of sitting during weekends was associated with lower mental well-being (p < 0.05). Similarly, more sitting while travelling at weekends was linked to lower work productivity (p < 0.05). In highly active employees, higher sitting times on work days and occupational sitting were associated with decreased mental well-being (p < 0.05). Higher sitting times while travelling on weekend days was also linked to lower work productivity in the highly active (p < 0.05). No significant associations were observed in low active employees.

**Conclusions:**

Employees’ PA levels exerts different influences on the associations between sitting time, mental well-being and work productivity. The specific associations and the broad sweep of evidence in the current study suggest that workplace PA strategies to improve the mental well-being and productivity of all employees should focus on reducing sitting time alongside efforts to increase PA.

## Background

Combining sitting reduction strategies with efforts to increase physical activity (PA) are important and complementary public health priorities [1-8]. In a recent meta-analysis, each additional hour of daily sitting – in adults who sat for >7 hours/day - increased risk in all-cause mortality by 2% [4]. The risk of dying from all causes increased to 5% for those who were also inactive, suggesting that PA may offer some protection against the harm of prolonged sitting time.

While the chronic disease benefits of sitting less and being more active are increasingly well documented [1-8], and associations observed between PA and mental well-being and work performance/productivity improvements [9-13], little is known about how sitting time influences these important workplace indices. Instead, existing research has explored associations between sitting time and markers of mental health, such as depressive symptoms, rather than well-being [14]. Further, what is known has only addressed non-occupational sitting time [15]. Recently, a small study of Australian office employees (n = 108) identified that more time spent sitting before and after work was associated with lost work productivity (odds ratio =2.58; 95% CI: 1.08 to 6.20) [16]. This study used an objective indicator of sitting time (accelerometers) to explore relationships with ‘on the job’ productivity indicators; interactions between behaviors were not assessed.

Given a limited evidence base, research is required to investigate the potential interactions between sitting time and PA, relative to mental well-being and work productivity. Such formative research will be valuable for developing interventions targeting specific employee behaviors that improve both health and efficiency-related outcomes. Consequently, this study examined relationships between self-reported sitting time, PA, mental well-being and work productivity in a sample of Spanish office employees.

## Methods

### Participants

Following ethics clearance, around 2,500 emails were sent to academic and administrative employees at each of four Spanish universities in Galicia, the Basque Country and Catalonia (×2). Emails invited employees to participate in a workplace PA program to increase step counts and reduce occupational sitting time. Respondents to this initial email (n = 704) were asked to complete an on-line survey (April- December 2010) prior to intervention. Informed consent was provided during survey completion. The study was approved by the following ethics committee of each university: Ethics Committee of the Faculty in Psychology, Education and Sport Sciences (University Ramon Llull); Research Commission of University of Vic; Ethics Committee of Clinical Research in Conselleria de Sanidad (CEIC; Xunta de Galicia); Ethics Committee of Applied Research in Human Beings (CEISH/GIEB; University of the Basque Country).

### Measures

A 22-item survey assessed socio-demographic variables (age, gender, weight, height and occupation [academic or administrator], PA levels [17], sitting time [18], mental well-being [19] and work productivity loss [20]. For PA, the *International Physical Activity Questionnaire* (IPAQ) short form assessed walking, moderate and vigorous intensity PA [17]. The IPAQ short form shows good reliability (Spearman’s ρ = 0.80) and moderate criterion validity with accelerometers (Spearman’s ρ = 0.30) in the general [17] as well as Catalan and Spanish populations [21].

Time spent in these activities was combined to show the volume of activity relative to energy expenditure (Metabolic Equivalent Units - METs), yielding a score in weekly MET-minutes. Employees were classified into either low (≥599 MET-minutes/week), moderate (at least 600–2,999 MET-minutes/week) or high (3,000+ MET-minutes/week) PA categories.

A seven-day total and domain-specific sitting questionnaire assessed weekly sitting time (minutes/day) at work and while travelling [18]. These domains were targeted within a workplace PA intervention that aimed to reduce sitting time (i) at work and (ii) while commuting. This questionnaire has high validity and reliability in the adult population for weekday sitting time at work (r = 0.69-0.74), while it is lower for weekend days across all domains (r = 0.23-0.74) [18]. Forward-backward translation into Catalan and Spanish identified linguistic equivalence [22].

The Warwick-Edinburg Mental Wellbeing Scale (WEMWBS) assessed positive mental well-being (positive functioning, happiness and subjective wellbeing) over the previous two weeks [19]. The 14-item scale has five response categories; 1 (“None”) to 5 (“All the time”). Responses are summed to identify the final score, 14–70, indicating low to high positive mental well-being. WEMWBS shows high internal reliability (Cronbach’s alpha = 0.93) and one week test-retest reliability (r = 0.97) in the Spanish population [23].

The *Work Limitations Questionnaire* (WLQ) was used to assess performance and the degree to which health problems interfered with the ability to perform job roles [20]. Spanish [24] and Catalan [25] versions of the WLQ have been developed and validated. In the WLQ, respondents self-report levels of difficulty in performing 25 specific job roles across four scales, with scores expressed as an average of responses. The 5-item “Time Scale” addresses difficulty in scheduling demands. For the “Mental-Interpersonal Scale” six items cover difficulty performing cognitive tasks involving the processing of sensory information and interacting with others on-the-job. The “Output Scale” has five items exploring limitations in meeting demands for quantity, quality and timeliness of completed work. The nine-item “Physical Scale” assesses ability to perform job tasks that involve bodily strength, movement, endurance, coordination and flexibility.

Sub-scales scores are transformed to a 0–100 continuum to represent the amount of time in the previous two weeks affected by limited on-the-job performance (from low to high rate of difficulty). These scales estimate work loss, known as the WLQ index [20], which is the weighted sum of the scores from the WLQ scales. In the present study, the WLQ index was calculated by summing the scores of three WLQ scales; the “Physical Scale” was excluded from the current analyses as it was not relevant to these job roles.

### Analyses

Data on key outcome variables were described using frequencies (percentage), means (standard deviation) and medians (interquartile range). Bivariate linear regression analyses assessed associations between self-reported sitting time (total and domain specific), PA, mental well-being and work productivity. The model was adjusted for demographics and stratified by PA level introducing an interaction term between PA level (low, moderate or highly active) [17] and sitting time into a multivariate regression model. Significance was set at p < 0.05 and analyses performed using Strata software, version 12.

## Results

Five-hundred and fifty-seven university office employees completed the survey, giving a response rate of 79% (557/704) from the initial respondents. Table 1 shows descriptive baseline data on the main variables as well as gender, mean age, mean body mass index, universities and staff occupation. Compared to males, females averaged 2.09 points lower on the WEMWBS scale indicating lower mental well-being (p < 0.05; Table 2).

**Table 1:** **Baseline data on the main outcomes and socio-demographic variables**

	**N = 557**
**Gender**, n (%)	
Male	215 (38.7)
Female	314 (61.3)
**Age**, mean (SD)	42 (9)
**Body Mass Index** (kg/m^2^), mean (SD)	24.86 (10.82)
**University**, n (%)	
Vic (Catalonia)	110 (19.8)
Basque Country	112 (20.1)
Ramon Llull – Blanquerna (Catalonia)	73 (13.1)
Vigo	261 (46.9)
**Occupation**, n (%)	
Academic Staff	340 (63.4)
Administrative Staff	196 (36.6)
**Physical Activity** (MET-minutes/week), median (interquartile range)	2,742 (1,238 - 4,921)
**Physical Activity** ^**1**^ (MET-minutes/week), n (%)	
Low	169 (31.5)
Moderate	151 (28.1)
High	217 (40.4)
**Mental Well-Being at work (WEMWBS)** ^2^, mean (SD)	52.6 (7.1)
**Presenteeism (WLQ)** ^3^, median (interquartile range)	
Time scale^4^	15 (5–25)
Mental-Interpersonal scale^5^	17 (8–28)
Output scale^6^	21 (8–29)
**% of work productivity loss (WLQ Index Score)** ^7^, median (interquartile range)	4.5 (2.5 - 6.6)
**SITTING**	
Time spent sitting at work (min/day), mean (SD)	287 (147)
**Time spent sitting traveling to and from places** (min/day), mean (SD)	
Weekdays	72 (48)
Weekend days	50 (48)
**Total time spent sitting (min/day)**, mean (SD)	
Weekdays	383 (209)
Weekend days	322 (186)

SD: Standard Deviation.

^1^High category: achieving a minimum total physical activity of at least 3,000 MET-minutes/weeks.

Moderate category: achieving a minimum total physical activity of at least 600 MET-minutes/weeks.

Low category: Individuals who do not meet criteria for categories 2 or 3.

^2^Warwick-Edinburgh Mental Well-being Scale (WEMWBS): The minimum score is 14 and the maximum is 70. Higher scores indicate better positive mental well-being.

^3^Each scale score indicates the percentage of time in the previous two weeks when the respondent was limited in performing a specific dimension of job tasks (from low to high rate of difficulty in performing job demands). The minimum score is 0 (limited none of the time) to 100 (limited all of the time).

^4^Five items addressing difficulty in scheduling demands.

^5^ Six items covering difficulty performing cognitive tasks at work.

^6^Five items addressing decrements in the ability to meet demands for quantity, quality and timeless of completed work.

^7^A percentage estimate of work loss based on the weighted sum of the scores from the WLQ scales.

**Table 2:** **Associations between mental well-being, work productivity loss and the scales for presenteeism with sitting time, PA and socio-demographic characteristics**

	**Mental Well-Being at work (WEMWBS)** ^**1**^	**WLQ Index Score** ^**2**^ **% of lost work productivity**	**Presenteeism (WLQ)** ^**3**^ **Time scale** ^**4**^	**Presenteeism (WLQ) Mental-Interpersonal scale** ^**5**^	**Presenteeism (WLQ) Output scale** ^**6**^
**Gender**					
Male	1	1	1	1	1
Female	−2.09 (−3.33, −0.85)*	0.22 (−0.60, 1.05)	2.45 (−1.29, 6.20)	2.37 (−1.44, 6.18)	−0.22 (−4.30, 3.87)
**Age**	0.05 (−0.02, 0.11)	0.002 (−0.041, 0.045)	−0.10 (−0.30, 0.09)	−0.09 (−0.29, 0.10)	0.08 (−0.13, 0.30)
**Body Mass Index** (kg/m^2^)	−0.02 (−0.07, 0.04)	0.033 (0.004, 0.063)*	0.15 (0.01, 0.29)*	0.16 (0.02, 0.31)*	0.13 (−0.03, 0.28)
**Occupation**					
Academic Staff	1	1	1	1	1
Administrative Staff	−0.22 (−1.46, 1.03)	−0.39 (−1.18, 0.39)	−0.29 (−3.86, 3.27)	−1.70 (−5.46, 2.05)	−4.39 (−8.45, −0.33)*
**Physical Activity** (MET-minutes/week)^7^	0.66 (0,09, 1,22)*	−0.50 (−0.91, −0.09)*	−1.47 (−3.32, 0.38)	−1.14 (−3.00, 0.72)	−3.25 (−5.25, −1.25)*
**Physical Activity** (MET-minutes/week)					
Physical activity low level	1	1	1	1	1
Physical activity moderate level	0.53 (−1.26, 2.31)	−1.21 (−2.16, −0.26)*	−7.31 (−11.64, −2.99)*	−4.84 (−9.28, −0.41)*	−4.69 (−9.50, 0.11)
Physical activity high level	2.33 (0.63, 4.02)*	−1.71 (−2.68, −0.75)*	−7.88 (−12.24, −3.52)*	−7.27 (−11.76, −2.77)*	−7.70 (−12.52, −2.88)*
**SITTING** ^8^					
**Time spent sitting at work** (min/day)	−0.004 (−0.072, 0.064)	0.05 (−0.009, 0.1)	0.17 (−0.08, 0.42)	0.25 (−0.02, 0.52)	0.30 (−0.01, 0.59)*
**Time spent sitting travelling** (min/day)					
Weekdays	0.02 (−0.17, 0.20)	−0.002 (−0.13, 0.12)	0.15 (−0.41, 0.72)	−0.03 (−0.61, 0.55)	−0.11 (−0.73, 0.50)
Weekend days	−0.18 (−0.37, 0,01)*	0.14 (0,01, 0.27)*	0.70 (0.12, 1.29)*	0.75 (0.16, 1.34)*	0.62 (−0.01, 1.26)*
**Total sitting time** (min/day)					
Weekdays	0.02 (−0.03, 0.06)	0.02 (−0.02, 0.07)	0.11 (−0.08, 0.31)	0.13 (−0.06, 0.33)	0.11 (−0.10, 0.32)
Weekend days	−0.10 (−0.14, −0.05)*	0.01 (−0.03, 0.05)	0.09 (−0.11, 0.28)	0.14 (−0.06, 0.33)	0.16 (−0.05, 0.37)

*p < 0.05.

^1^ Warwick-Edinburgh Mental Well-being Scale (WEMWBS): Scores range 14 to 70. Higher scores indicate better positive mental well-being.

^2^A percentage estimate of work loss based on the weighted sum of the scores from the Work Limitations Questionnaire (WLQ) scales.

^3^The estimated percentage of time in the previous two weeks spent feeling limited in performing a specific dimension of job tasks (rated from low to high difficulty).

^4^Five items addressing difficulty in scheduling demands.

^5^Six items cover difficulty performing cognitive tasks involving the processing of sensory information and a person’s problems interacting with people on-the-job.

^6^Five items address decrements in the ability to meet demands for quantity, quality and timeless of completed work.

^7^Coefficient (95% Confidence Interval) corresponding to the physical activity logarithm.

^8^The coefficients of the different domains of sitting correspond to an increase of 15 min/day.

A higher body mass index was also significantly associated with greater losses in work performance (p < 0.05; Table 2) and an increased difficulty in achieving scheduling demands, performing cognitive tasks and interacting with others on the job (p < 0.05; Table 2). No significant associations were identified between body mass index, mental well-being or meeting demands for quantity and quality of completed work.

**Figure 1:**
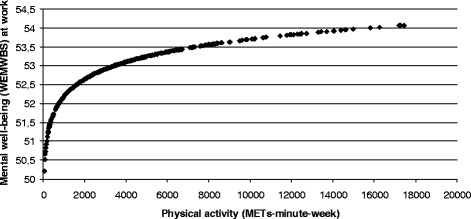
**Significant positive non-linear association between physical activity (METs-minute-week) and mental well-being (WEMWBS) at work.** ^1^Warwick-Edinburgh Mental Well-being Scale (WEMWBS): The minimum score is 14 and the maximum is 70. Higher scores mean better positive mental well-being.

**Figure 2:**
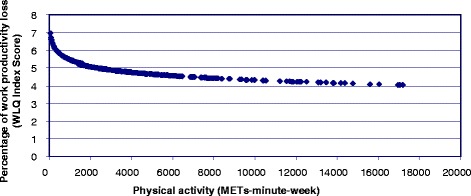
**Significant negative non-linear association between physical activity (METs-minute-week) and percentage of work productivity loss (WLQ Index Score).** ^1^Estimate of the percentage of work loss based on the weighted sum of the scores from the Work Limitations Questionnaire scales.

Higher volumes of PA (MET-minutes/week) were positively related to better mental well-being (p < 0.05; Table 2). While the least active employees reported the lowest WEMWBS scores, employees who did more PA reported higher scores (Figure 1). As PA rose from zero METs-minute/week, average WEMWBS scores rose sharply. However, WEMWBS averages were similar with higher levels of PA (Figure 1). Higher PA (MET-minutes/week) was also beneficially associated with the percentage of lost work performance (p < 0.05; Table 2). The least active employees reported the greatest percentages of lost productivity compared to the most active employees (Figure 2). As PA rose from zero METs-minute/week, the percentage of lost work performance was sharply reduced; the lowest level of lost work performance was reported among employees doing most PA (Figure 2). A consistent pattern showed that progressively more PA was inversely linked to the time employees spent feeling limited in their capacities (Table 2). This was shown for (i) scheduling demands (linked to a 22.60%, 15.86% and 14.67% of time feeling limited for the low, moderate and high PA categories respectively), (ii) performing mental-interpersonal tasks (24.42%, 20.16% and 17.12%) and (iii) delivering outputs (28.16%, 23.73% and 21.24%) (Table 2). Each category of PA was linked to a smaller, but still progressive, percentage estimate for lost work productivity; 5.99%, 4.95% and, 4.36% (Table 2).

**Figure 3:**
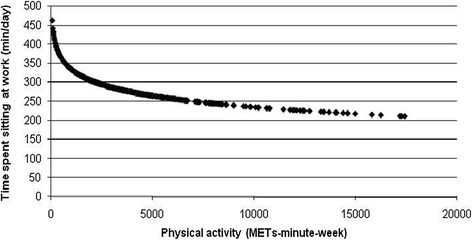
**Significant negative non-linear association between physical activity (METs-minute-week) and occupational sitting time.**

**Figure 4:**
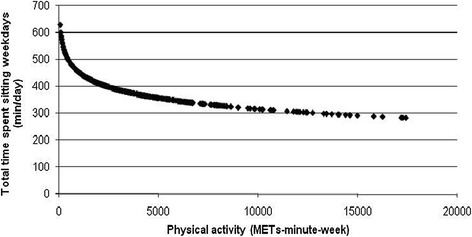
**Significant negative non-linear association between physical activity (METs-minute-week) and total time spent sitting during weekdays.**

**Figure 5:**
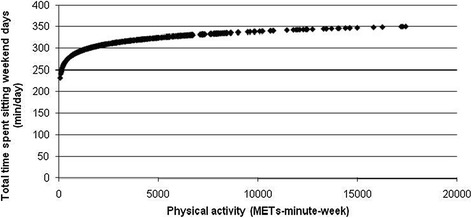
**Significant positive non-linear association between physical activity (METs-minute-week) and total time spent sitting on weekend days.**

Higher volumes of PA were also associated with spending less time sitting at work and throughout the working day (p < 0.05; Figures 3 and 4). While the least active employees reported higher times of occupational sitting and daily sitting during weekdays, employees engaged in high volumes of PA reported the least time sitting on both domains (Figures 3 and 4). As PA MET minutes/week rose from zero, the average minutes spent sitting at work and during working days reduced. However, the rate of decrease on occupational and total weekday sitting time lessened when PA was high (Figures 3 and 4). Contrarily, higher volumes of PA were significantly associated with spending more time sitting at weekends (p < 0.05; Figure 5). As PA increased from zero METs-minute/week, the average of minutes spent sitting at weekends increased more sharply than when PA was higher (Figure 5). Higher volumes of PA were also significantly associated with less time spent sitting while travelling during weekends and weekdays (p < 0.05). While low active employees spent an average of 62 minutes/day sitting during weekend travel, the comparable average for moderately and highly active employees was 45 (62.38 to 17.61) minutes/day. Similarly, for weekday travelling low active employees averaged 77 minutes/day sitting compared to 59 (77.03 to 18.38) minutes/day for the moderately active (p < 0.05).

Two domains of sitting time showed significant negative linear associations with positive mental well-being (p < 0.05; Table 2). Greater levels of sitting in weekend travelling and total weekend sitting time were associated with lower mental well-being; sitting 30 extra minutes a day in each domain was linked to a reduction of 0.6% and 0.4% respectively; 0.36 and 0.20 points in the WEMWBS score respectively (p < 0.05; Table 2). No significant associations were found between weekday occupational and total sitting time for mental well-being.

Time spent sitting during weekend travelling also showed an inverse relationship with work productivity (p < 0.05; Table 2). More sitting during weekend travelling was related to greater difficulties in meeting job demands (every extra 30 minutes/day was linked with an additional 1.4% difficulty in meeting job scheduling demands, a 1.5% increase in difficulty performing cognitive tasks or tasks that involved interacting with others and a 1.2% increase in the difficulty of meeting demands for quantity, quality and timeless of completed work). Greater levels of sitting while travelling at weekends were also linked to lower overall work productivity (each additional block of 30 minutes/day was linked to a reduction of 0.3% (p < 0.05; Table 2). There were no significant associations between productivity and occupational or total sitting time.

In highly active employees, greater levels of sitting at work, throughout the work day and while travelling during weekend was related to lower mental well-being (for each increment of 30 minute/day was related to a reduction of 0.6%, 0.36% and 1.9% respectively (0.34, 0.2 and 1.06 points in the WEMWBS scores; Table 3). This association was not significant for inactive employees (Table 3). Greater levels of sitting in weekend travel was also linked to lower work productivity in the highly active (each additional 30 minutes was associated with a 0.8% reduction). Among inactive employees, no domain of work productivity was linked to higher sitting time.

**Table 3:** **Interaction between PA levels and sitting time relative to mental well-being, work productivity loss and the scales for presenteeism adjusted for demographics**

	**Mental Well-Being at work (WEMWBS)** ^**1**^	**WLQ Index Score** ^**2**^ **% of lost work productivity**	**Presenteeism (WLQ)** ^**3**^ **Time scale** ^**4**^	**Presenteeism (WLQ) Mental-Interpersonal scale** ^**5**^	**Presenteeism (WLQ) Output scale** ^**6**^
**Time spent sitting at work** (min/day)^7^					
Physical activity low level	−0.10 (−0.32, 0.13)	0.004 (−0.12, 0.13)	0.09 (−0.46, 0.63)	−0.02 (−0.61, 0.56)	0.19 (−0.78, 0.41)
Physical activity moderate level	−0.13 (−0.27, 0.01)	0.03 (−0.06, 0.11)	0.03 (−0.39, 0.32)	0.20 (−0.19, 0.58)	0.24 (−0.19, 0.67)
Physical activity high level	−0.17 (−0.31, −0.03)*	0.07 (−0.02, 0.16)	0.32 (−0.06, 0.69)	0.32 (−0.11, 0.75)	0.33 (−0.13, 0.79)
**Time spent sitting travelling to and from places on weekdays** (min/day)					
Physical activity low level	0.21 (−0.16, 0.59)	−0.17 (−0.41, 0.06)	−0.59 (−1.77, 0.59)	−0.58 (−1.64, 0.48)	−1.00 (−2.24, 0.24)
Physical activity moderate level	0.02 (−0.35, 0.40)	−0.04 (−0.27, 0.18)	−0.28 (−1.17, 0.60)	0.01 (−0.99, 1.01)	−0.07 (−1.18, 1.04)
Physical activity high level	−0.07 (−0.36, 0.21)	0.08 (−0.10, 0.26)	0.63 (−0.16, 1.42)	0.06 (−0.88, 1.00)	−0.11 (−1.17, 0.95)
**Time spent sitting travelling to and from places on weekend days** (min/day)					
Physical activity low level	0.22 (−0.15, 0.58)	−0.15 (−0.38, 0.07)	−0.73 (−1.88, 0.42)	**−**0.44 (−1.47, 0.59)	−0.80 (−1.95, 0.35)
Physical activity moderate level	−0.23 (−0.62, 0.15)	−0.05 (−0.28, 0.19)	−0.24 (−1.21, 0.73)	0.22 (−0.81, 1.24)	−0.12 (−1.30, 1.07)
Physical activity high level	−0.53 (−0.84, −0.22)*	0.40 (0.21, 0.59)*	1.83 (0.99, 2.66)*	1.61 (−0.59, 2.63)*	1.47 (0.40, 2.54)*
**Total time spent sitting on weekdays** (min/day)					
Physical activity low level	−0.02 (−0.14, 0.10)	−0.03 (−0.11, 0.05)	−0.15 (−0.57, 0.27)	−0.04 (−0.38, 0.31)	−0.21 (−0.59, 0.17)
Physical activity moderate level	−0.14 (−0.26, −0.02)*	0.02 (−0.05, 0.09)	0.03 (−0.27, 0.33)	0.16 (−0.15, 0.47)	0.14 (−0.23, 0.50)
Physical activity high level	−0.10 (−0.21, −0.002)*	0.06 (−0.01, 0.12)	0.28 (−0.01, 0.58)	0.19 (−0.16, 0.54)	0.13 (−0.23, 0.50)
**Total time spent sitting on weekend days** (min/day)					
Physical activity low level	−0.10 (−0.21, 0.001)	−0.01 (−0.07, 0,06)	0.06 (−0.29, 0.41)	0.12 (−0.19, 0.42)	0.06 (−0.26, 0.39)
Physical activity moderate level	−0.03 (−0.15, −0.09)	−0.03 (−0.04, 0.10)	0.16 (−0.13, 0.45)	0.09 (−0.23, 0.41)	0.25 (−0.11, 0.61)
Physical activity high level	−0.03 (−0.15, 0.10)	−0.02 (−0.10, 0.06)	−0.12 (−0.48, 0.24)	0.03 (−0.40, 0.47)	−0.15 (−0.58, 0.27)

*p < 0.05.

^1^Warwick-Edinburgh Mental Well-being Scale (WEMWBS): Scores range 14 to 70. Higher scores mean better positive mental well-being.

^2^A percentage estimate of work loss based on the weighted sum of the scores from the Work Limitations Questionnaire (WLQ) scales.

^3^A percentage estimate of time in the previous two weeks spent feeling limited in performing a specific dimension of job tasks (rated from low to high difficulty).

^4^Five items addressing difficulty in scheduling demands.

^5^Six items cover difficulty performing cognitive tasks involving the processing of sensory information and a person’s problems interacting with people on-the-job.

^6^Five items address decrements in the ability to meet demands for quantity, quality and timeless of completed work.

^7^The coefficients of the different domains of sitting correspond to an increase of 15 min/day.

## Discussion

This study examined the associations between sitting time and PA, with mental well-being and work productivity in 557 office employees. Uniquely, the study addresses cross-sectional differences in how indices of sitting, alone or in combination with markers of PA, relate to mental well-being and productivity. Given the need to develop workplace PA interventions that target employees’ health related efficiency outcomes, this study provides novel insights of the interactive relationships between sitting time and PA. This evidence contributes to a better understanding of how targeting both behaviors can potentially benefit the mental well-being and productivity of office employees.

The main finding of the present study indicated that employees’ PA levels exerted different influences on the associations between sitting time, mental well-being and work productivity. Previous research has reported adverse associations between prolonged sitting time and well-being in adults [15,26]. However, these studies have focused on leisure-time sitting (i.e. television viewing and screen-based sitting) rather than on investigating how employees’ PA levels might interact on the results of occupational sitting time and subsequent effects on mental well-being. While several studies have examined joint associations between PA and sitting time with physical health outcomes [27,28], few have investigated the interactive effects of PA and sitting time relative to mental well-being.

Rosenkranz et al. [29] identified that PA was positively associated with excellent overall health (OR 2.22, 95% CI = 2.20. 2.47) and quality of life (OR = 1.13, 95% CI = 1.09, 1,18), interactions between PA and sitting time were not statistically significant. Similarly, Södergren et al. [30] addressed the relationship between leisure PA and sitting time to examine their associations with good self-reported health. No associations between sitting time and self-rated health were identified using multivariate analysis. However, both of these studies [29,30] measured total daily sitting time by asking participants to report total hours per day usually spent sitting (using IPAQ short and long forms). Neither investigated the joint associations between PA and different domains of sitting time relative to mental well-being.

In our study, spending more time sitting at work and during workdays was linked to lower mental well-being in the highly active employees but not in their inactive counterparts. A possible explanation could be the relationship identified in our sample between PA and both sitting time domains. While highly active employees averaged 3.5 hours sitting at work and 5.4 hours/day sitting from Mondays to Fridays, their low active counterparts averaged 5.15 hours/day and 7.11 hours/day sitting respectively. For highly active employees, increasing sitting time may indicate a decline healthy daily behavior, with negative consequences for their mental well-being. Even though no threshold for sitting time has been linked to diminished mental well-being, previous research has identified that sitting for more than 7 hours/day was associated with an increased likelihood of depressive symptoms in women [31]. Since adopting one healthy lifestyle behavior can facilitate adopting another [32], and similarly for negative behavior, future research should examine how changes to the domains of sitting time relate to mental well-being in highly active employees.

For the same group, higher volumes of time spent sitting travelling at weekends were associated with both poorer work performance and poorer mental well-being. To our knowledge, no previous studies have investigated the joint associations between PA with total and specific domains of sitting time relative to employees’ work performance. Our results indicated that time spent sitting while travelling during non-working days influenced employees’ work productivity and, that PA levels exerted an influence on this association. Again, this may be explained by the relationship identified in our sample between PA and sitting time while travelling at weekends; with highly active employees sitting 17 minutes/day less while travelling on Saturday and Sundays than their inactive counterparts. While a recent systematic review [33] identified that the most commonly assessed subtypes of sitting domains were TV viewing, total sitting, general screen and occupational sitting time - with each being associated with lower levels of PA – few studies have examined how specific sitting domains during non-working days are influenced by PA levels or vice versa. Even less is known about how this relates to work-related issues such as work productivity or performance. Our results are partly consistent with previous research that indicates that highly active employees sit less at work and also outside work [34], including commuting, even though the previous study only referenced commuting on weekdays. However, the associations found in our sample between PA and total sitting time during non-working days suggests a different pattern of sedentary and PA behaviors during non-working days. This change in patterns is consistent with a previous study indicating that the time periods of 06:00–07:00 and 17:00–19:00, which are typically outside normal working hours, represent the periods when moderate-to-vigorous PA is significantly higher in work days than non-working days [35]; being at work from 09:00–17:00 clearly influences employees’ sedentary and PA patterns during the workdays [35]. Furthermore, previous research has suggested that engaging in sitting behaviors is related to having more leisure time, which mainly happens on the weekends of working adults [36] and that sitting time during non-working days is explained by different correlates (i.e. home and neighborhood factors) than working days [36]. Future research should investigate the effects sedentary patterns on non-working days have on work productivity as well as mental well-being.

Finally, it should be pointed out that more sitting time domains were related to mental well-being than to work productivity. Nonetheless, mental well-being has been associated with work productivity and other work-related outcomes (i.e. job stress), indicating that specific domains of sitting time could also indirectly influence work productivity. A recent longitudinal study identified that employees in the low well-being segment reported over 3 times the level of work productivity loss than those in the high well-being segment [37]. Over a year, changes in well-being were significantly associated with positive changes in employees’ productivity [37]. Additionally, levels of positive mental well-being reduce as work stress increases [38]; work stress is one of the most commonly reported causes of work-related illness and loss of work performance [38].

This study has several important limitations. As a cross-sectional study, it is not possible to establish cause-effect relationships between sitting time, PA, mental well-being and productivity. Furthermore, the data can only indicate associations; studies are needed to address the directionality of these associations. More PA and less sitting may be result of better mental health and performance. However, descriptive analyses are essential for documenting the potential benefits of health promotion initiatives [39] for employees. This descriptive study provides a valuable baseline for developing workplace interventions aimed at improving employees’ well-being and work productivity through sitting behavior *and* PA. It is also important to recognize that our findings are specific to office employees (highly educated middle-age men and women) who showed an interest to participate in a workplace PA program (Walk@WorkSpain). Ongoing research should focus on more heterogonous samples of office employees. In addition, sitting time and PA were measured by self-report. Estimates of workplace sitting are generally higher when measured using objective devices than when measured by self-report [40]. Furthermore, self-report estimates of work performance/productivity and mental well-being have the potential to contain error. However, in the current study these domains were measured by using two scales with high validity and reliability. Objective measures of sitting time are needed to generate deeper insights into the relationship between total and specific sitting domains with employee’s well-being and productivity.

## Conclusion

Our findings present a strong rationale, based on consistent associations, for combining sitting time reduction strategies with efforts to increase PA in interventions aimed at improving office employees’ well-being and productivity. The study identified distinctive associations depending on pre-existing PA levels. In highly active employees, less total sitting time and occupational sitting on work days was associated with better mental well-being and work performance. In inactive employees, higher levels of PA were related to better mental health and higher performance estimates. This study also suggests that workplace PA programs promoting “sitting less” in different domains –including weekends - may beneficially impact work productivity and mental well-being. Future research should investigate the impact of workplace sitting time reduction strategies on work productivity and mental well-being among employees engaged in different levels of pre-existing PA.
